# Specific EEG Sleep Pattern in the Prefrontal Cortex in Primary Insomnia

**DOI:** 10.1371/journal.pone.0116864

**Published:** 2015-01-22

**Authors:** Joy Perrier, Patrice Clochon, Françoise Bertran, Colette Couque, Jan Bulla, Pierre Denise, Marie-Laure Bocca

**Affiliations:** 1 Normandie Univ, Caen, 14032, France; 2 UNICAEN, COMETE, Caen, 14032, France; 3 INSERM, U 1075 COMETE, Caen, 14032, France; 4 CHU de Caen, Department of Clinical Physiology, Caen, 14033, France; 5 INSERM, U1077, Caen, 14074, France; 6 UNICAEN, UMR-S1077, Caen, 14074, France; 7 Ecole Pratique des Hautes Etudes, UMR-S1077, Caen, 14074, France; 8 Department of Mathematics, University of Bergen, P.O. Box 7800, 5020 Bergen, Norway; Oasi research institute, ITALY

## Abstract

**Objective:**

To assess the specific prefrontal activity in comparison to those in the other main cortical areas in primary insomnia patients and in good sleepers.

**Methods:**

Fourteen primary insomnia patients and 11 good sleepers were included in the analysis. Participants completed one night of polysomnography in the sleep lab. Power spectra were calculated during the NREM (Non-rapid eyes movements) and the REM (Rapid eyes movements) sleep periods at prefrontal, occipital, temporal and central electrode positions.

**Results:**

During the NREM sleep, the power spectra did not differ between groups in the prefrontal cortex; while primary insomnia patients exhibited a higher beta power spectrum and a lower delta power spectrum compared to good sleepers in other areas. During the REM sleep, the beta1 power spectrum was lower in the prefrontal cortex in primary insomnia patients compared to good sleepers; while no significant difference between groups was obtained for the other areas.

**Conclusions:**

The present study shows a specific prefrontal sleep pattern during the whole sleep period. In addition, we suggest that primary insomnia patients displayed a dysfunction in the reactivation of the limbic system during the REM sleep and we give additional arguments in favor of a sleep-protection mechanism displayed by primary insomnia patients.

## Introduction

Between 5 and 30% of the general population has complaints of insomnia which make this pathology one of the most prevalent sleep disorders [1–3]. It is a public health problem as the annual direct and indirect costs were estimated to $1 253 in the United-States [4] and to $5,010 per year in the province of Quebec (Canada) [5]. Among the different types of insomnia defined by the DSMIV, the primary insomnia (PI) is a complaint of non restorative and/or insufficient sleep with difficulties of initiating and maintaining sleep, and early morning awakenings associated with daytime consequences, without comorbidity. Using this definition, PI represents 2–4% of the general population [6].

Despite the socioeconomic burden and the daytime consequences of insomnia [5], this pathology and its sleep characteristics are poorly understood [7,8]. Indeed, polysomnography (PSG) investigations in PI have not consistently revealed modifications in sleep architecture. Especially, PSG derived sleep variables produced far less pronounced differences to good sleepers than expected from the subjective estimates of the patients’ sleep quality [9]. A more fine-grained approach as the investigation of power spectra analyses (PSA) is considered more promising to better understanding the pathophysiology of PI [10]. A general finding in PSA is an increased beta activity during the NREM in PI patients compared to good sleepers [11–15], which reflects a cortical hyperarousal in PI [7,16]. In contrast to NREM findings, REM investigations have led to inconsistent results as significant differences in beta power spectrum between PI patients and good sleepers were found in two studies [11,13]; while no group’s differences was found in two other studies [12,15].

As previous sleep spectral analysis studies did not compare the cortical activity between various brain areas, no specific cortical activity during sleep in one area compared to the other has been already highlighted. Then, further PSA investigations will be useful to better understand the sleep spectral modifications that could occurred in specific cortical area in PI and then the physiopathology of this sleep disorder. Indeed, several neuropsychological studies have revealed that PI patients had impaired performance in tests involving the prefrontal cortex [17–20] and neuroimaging studies showed brain function and morphology alterations in the prefrontal cortex [21–23, respectively]. As the prefrontal cortex has a major role in mediating sleep physiology i.e deactivation during the transition between wake and NREM, increasing of this deactivation with the deepening of the NREM sleep and reactivation during the sleep [24–26], we can suppose that alterations or modifications in the prefrontal cortex could be present during the sleep period in PI patients. The objective of the current investigation was thus to assess the sleep EEG power spectra in PI patients and in good sleepers in four main cortical areas (temporal, central and occipital and prefrontal) and to compare the EEG power spectra in other areas (occipital, central and parietal) to those in the prefrontal area.

## Methods

### Participants

The data presented in this paper are part of a study whose results concerning behavioral (driving performance) data were recently published [27]. The polysomnographies were recorded the night before the driving evaluation for all participants. For details, twenty one PI patients and sixteen good sleepers were included. After inspection for spectral analysis, due to a strict artifact rejection (eyes, movements etc), all recordings could not been analyzed. Consequently, both sleep architecture and spectral analysis were here presented for the same participants remaining, i.e fourteen PI patients (5 men and 9 women; mean age = 47 ± 17; age range 24–74 years) and ten good sleepers (4 men and 6 women; mean age = 46 ± 15; age range = 23–64 years).

After a telephone interview, the participants from both groups had a medical interviewed by a sleep clinician to ascertain i) sleeping difficulties and diagnose DSM-IV insomnia for the insomniac patients’s group ii) good physical condition; the absence of sleep, alertness, neurological, cardiovascular, respiratory, hepatic, renal or metabolic disorders; presence of poor hygiene or habitual abnormal sleep patterns (i.e. night or shift work) for the good sleeper’s group.

The participants included in the insomnia group had to meet the following inclusion criteria according DSM-IV primary insomnia criteria: (a) presence of a subjective complaint of insomnia, defined as difficulty initiating (i.e., sleep onset latency (SOL), > 30 min) and/or maintaining (i.e., time awake after sleep onset > 30 min) sleep, (b) early awakening (i.e., < 6.5 h of sleep or waking up earlier than the desired wake time); (a) and (b) had to occur at least three nights per week; (c) insomnia duration of at least 6 months; (d) insomnia or its perceived consequences causing marked distress or significant impairment of occupational or social functioning (i.e, problem of concentration); and (e) presence of a subjective complaint of at least one negative daytime consequence attributed to insomnia (i.e, fatigue, mood disturbances).

The exclusion criteria were: (a) significant current medical or neurological disorder that could compromise sleep; (b) major psychopathology that could induce insomnia; (c) psychotropic or other medications consumption known to alter or induce sleep; (d) poor hygiene or habitual abnormal sleep patterns (e.g. night or shift work) and (e) another sleep disorders (assessed by polysomnography) such as sleep apnea (apnea-hypopnea index > 10), or periodic limb movements during sleep (myoclonic index with arousal >10). All insomnia and good sleepers participants were excluded if they i) had a current or past dependence on alcohol, opiates, benzodiazepines or any illicit drugs; ii) smoke more than five cigarettes per day; iii) drunk more than 28 units of alcohol per week or iv) consumed more than 150 mg of caffeine per day. All participants had normal or corrected to normal vision (visual acuity greater than or equal to 7/10).

The study was granted ethical approval by the Caen Northwest III ethics committee and by the Health Ministry (number DGS 2005/0388). Each participant gave written consent in accordance with the requirements of the committee.

### Polysomnography

A standard laboratory procedure was conducted in all participants for one experimental night.

Sleep was recorded using a polysomnography ambulatory monitoring (Medatec Dream) machine. A standard montage of polysomnography was used including 8 EEG channels (FP1, FP2 C3, C4, O1, O2, T3, T4, referenced on linked mastoid A1 and A2), 2 electrooculogram (EOG) channels and one submental electromyogram channel. This montage was complemented by recordings from the left and right anterior tibialis muscle, recordings of nasal/oral airflow, thoracic and abdominal effort, body position) and oximetry. All PSG were scored according to the standard criteria [28] by experienced sleep specialists (CC, FB).

The objectives measures of sleep included sleep onset latency (SOL, min), wake after sleep onset (WASO, min), total sleep time (TST, min), sleep efficiency (SE, %), total time (min), latencies (min), and percentage (%) of Stages 1, 2 and 3+4 and rapid eye movement (REM).

### Spectral analysis

The EEG data were filtered with a band pass filter of 0.16–70 Hz and sampled at 200 Hz. Computerized spectral analysis was performed with fast Fourier transformation (FFT) on the all-night ﬁltered EEG after elimination of epochs with artifacts (eye-movement, electrocardiogram, electromyogram or movement-related artifacts). Spectral analysis was performed on 5.12-s epochs and on the 8 channels recorded (FP1, FP2, C3, C4, O1, O2, T3, T4, referenced on linked mastoid A1 and A2) to investigate the difference in areas EEG power spectra. Before computing the FFT, the data were tapered with the Hamming window. The FFT was computed on artifact-free epochs. The FFT was realized for each sleep stage on the total number of epochs corresponding to the maximal number of artifact-free epochs observed in all subjects. The relative power spectrum was obtained in the following frequency bands: delta (1.5–4 Hz); theta (4–7.5 Hz); alpha (separated in alpha1, alpha2 and alpha3 and aggregated) (7.5–12.5 Hz); sigma (12.5–14 Hz); beta (separated in beta1, beta2 and beta3 and aggregated) (14–30 Hz). All night power averages were obtained for stage 2, stage 3–4 and REM sleep separately.

### Statistical analysis

Statistical analyses of EEG data were performed with SAS software (SAS Institute Inc., 9.3) and R (3.0.1) [29]. Polysomnography variables were compared by using a variance analysis with the GLM procedure with comparison between groups (PI patients versus good sleepers). For spectral analysis, we used an analysis of variance (ANOVA) with two factors, including group and areas (prefrontal versus other areas, where the latter correspond to mean of the three other areas). Post hoc comparisons between groups and areas for significant interactions were conducted with Tukey’s honest significance test to control for multiple comparisons. Moreover, we calculated effect sizes concerning group comparisons for the architecture analysis and concerning group comparisons and group by area interactions for the spectral analysis results using partial omega squared [30]. For the spectral analysis, only results of the group effect and the group by area interactions are described in the results section. The significance level was set at *p*<0.05.

## Results

Participant’s characteristics are given in Table 1 for each group.

**Table 1:** Baseline characteristics of participants.

Questionnaires	Insomnia patients (n = 17) Mean (SD)	Good sleepers (n = 11) Mean (SD)	*p* value
ISI	18.5 (4.80)	3.29 (1.38)	0.006**
PSQI	10.86 (2.67)	3.90 (2.38)	<0.001***
Horne and Ostberg	56.92 (10.24)	58.80 (9.80)	0.66
Age	47 (16.82)	44.2 (14.96)	0.68
Sex	5M/9F	4M/6F	/

Between-group analyses (unpaired *t* tests). Equality of variance correction was applied. SD: Standard Deviation, ISI: Insomnia Severity Index, PSQI: Pittsburg Sleep Quality Index.

**p <0.05;

***p<0.001.

### Polysomnography

Polysomnography results are presented in Table 2. Statistical results revealed that the sleep efficiency was lower and that the number of WASO was higher in PI patients compared to good sleepers.

**Table 2:** Sleep characteristics of participants.

Sleep parameters	Insomnia patients (n = 14) Mean (SD)	Good sleepers (n = 10) Mean (SD)	*p* value	ω²_p_ [CI 90%]
TSP (min)	457.26 (23.03)	4451.68 (48.50)	0.71	−0.037 [0.00; 0.13]
TST (min)	382.39 (544.71)	408.42 (47.97)	0.19	0.034 [0.00; 0.28]
SE (%)	83.73 (9.45)	90.05 (7.07)	0.071*	0.098 [0.00; 0.35]
REM Latency (min)	106.20 (38.85)	101.29 (40.50)	0.78	−0.040 [0.00; 0.11]
Number of WASO (>1min)	09.36 (4.83)	5.89 (2.56)	0.051*	0.12 [0.00; 0.37]
Stage 1 (% TST)	10.46 (4.90)	12.062 (3.35)	0.24	0.018 [0.00; 0.25]
Stage 2 (% TST)	43.44 (10.44)	43.89 (7.93)	0.91	−0.042 [0.00; 0.05]
SWS (% TST)	29.01(10.55)	26.00 (6.00)	0.42	−0.014 [0.00; 0.20]
REM (% TST)	17.09 (5.94)	17.15 (4.16)	0.85	−0.042 [0.00; 0.086]

*0.1>p>0.05; Between groups analysis using two-way ANOVA (GLM procedure), insomnia patients vs good sleepers.

TSP: Total Sleep Period, TST: Total Sleep Time, SE: Sleep efficiency, REM: Rapid Eye Movement, WASO: Waking After Sleep Onset, SWS: Slow Wave Sleep, SD: Standard Deviation, ω²_p_: partial omega squared.

### Spectral analysis

Significant results of the power spectral analysis for the group effects and the group by area interaction for both NREM sleep and REM sleep are summarized in Table 3. Statistical results for the post hoc comparisons are given in the text. To focus the statistical description to the spectral changes in PI compared to the good sleepers related to brain areas, only the significant group effects and the post-hoc corresponding to the significant group by area interactions are detailed for each sleep stage and each frequency band in the following section.

**Table 3:** Significant results of the power spectral analysis.

Stages	Bands	Group effect DF = 3		Group*Areas Effect DF = 1		ω²_p_ [CI 90%]	
		F	p	F	p	Group effect	Groups*Areas effect
Stage 2	Delta	0.02	0.895	4.55	0.039**	−0.021 [0.00; 0.03]	0.069 [0.003; 0.23]
	Beta	0.02	0.90	8.60	0.005**	−0.021[0.00; 0.028]	0.14 [0.29; 0.31]
Stage 3+4	Delta	3.56	0.066*	4.16	0.048**	0.051 [0.00; 0.21]	0.062 [0.00; 0.22]
	Alpha	6.48	0.015**	0.58	0.45	0.10 [0.014; 0.27]	−0.009 [0.00; 0.11]
	Sigma	5.95	0.019**	6.37	0.015**	0.093 [0.011]	0.10 [0.013; 0.27]
	Beta	1.29	0.26	11.65	0.0014**	0.0059 [0.00; 0.14]	0.18 [0.053; 0.36]
REM	Beta1	3.06	0.087*	3.66	0.062*	0.041 [0.00; 0.20]	0.05 [0.00; 0.21]

*0.1>p>0.05

**p<0.05

Groups effect, insomnia patients versus good sleepers

Groups*Areas effect, interaction between group effect and frontal versus others scalp areas effect

REM, Rapid Eye Movement

DF, Degree of Freedom, ω²_p_: partial omega squared.

**Figure 1:**
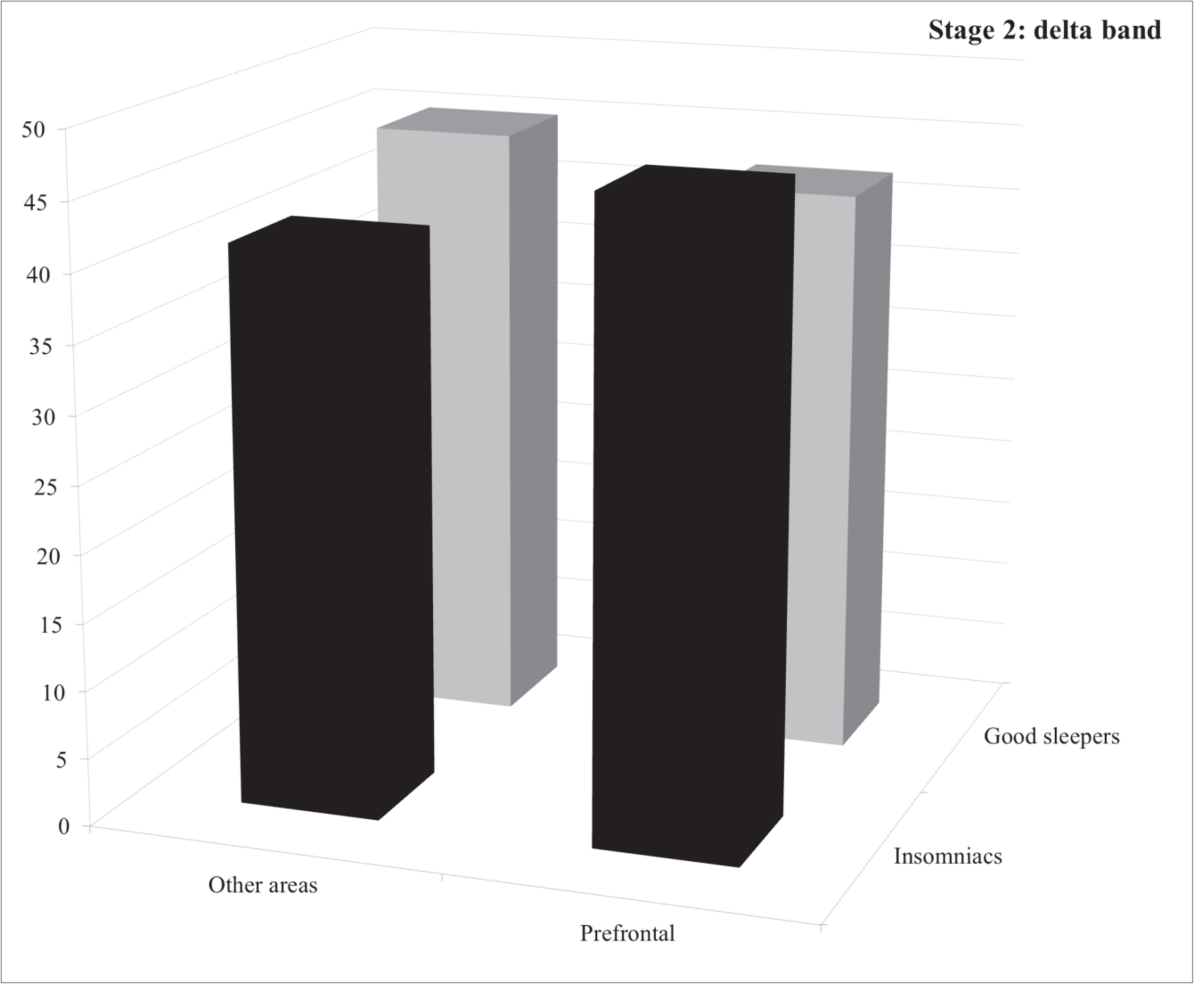
Relative power spectra for the delta band during stage 2 for both groups (insomnia patients and good sleepers) and each areas (others vs prefrontal). ** p<0.05.

**Figure 2:**
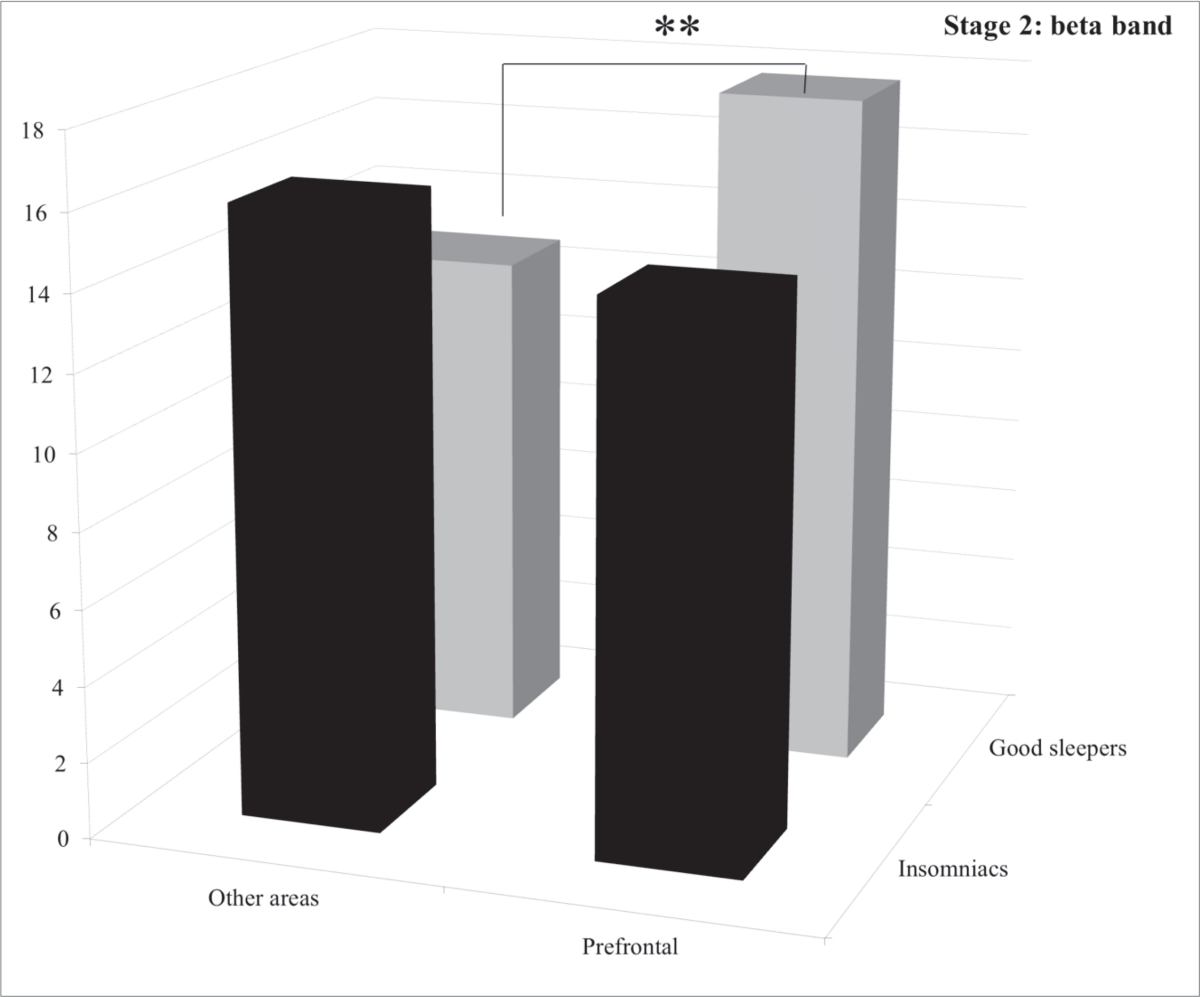
Relative power spectra for the beta band during stage 2 for both groups (insomnia patients and good sleepers) and each areas (others vs prefrontal). ** p<0.05.

**Stage 2.** For both the delta and the beta bands, statistical analysis revealed a group by area interaction but no significant difference between groups. For the delta band, post hoc comparisons revealed no significant difference both between groups and between areas (See Fig. 1). For the beta band, post hoc comparisons revealed that good sleepers displayed significantly less power spectrum in the “other areas” than in the frontal area (*p* = 0.030) (See Fig. 2).

**Figure 3:**
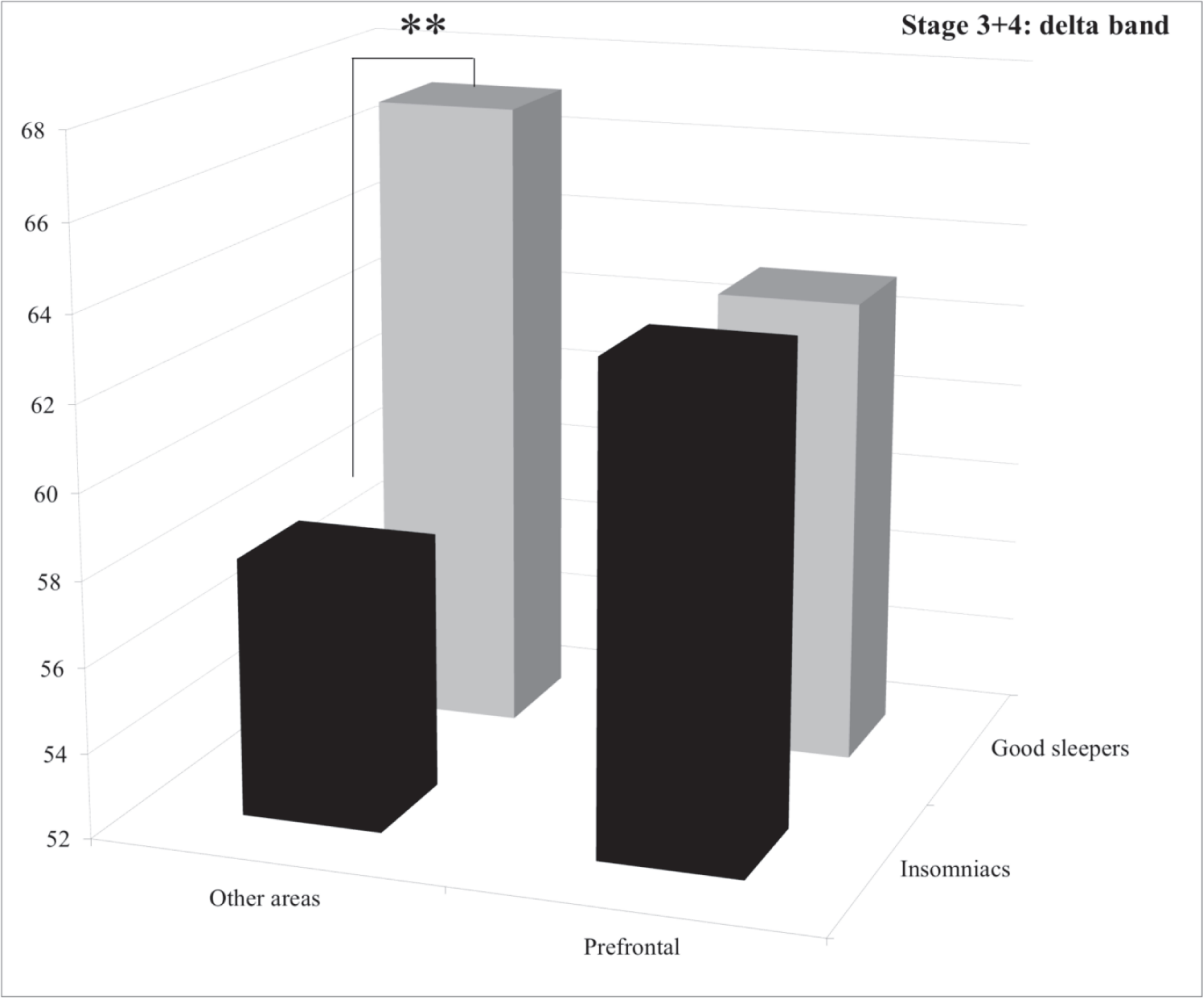
Relative power spectra for the delta band during stage 3+4 for both groups (insomnia patients and good sleepers) and each areas (others vs prefrontal). ** p<0.05.

**Figure 4:**
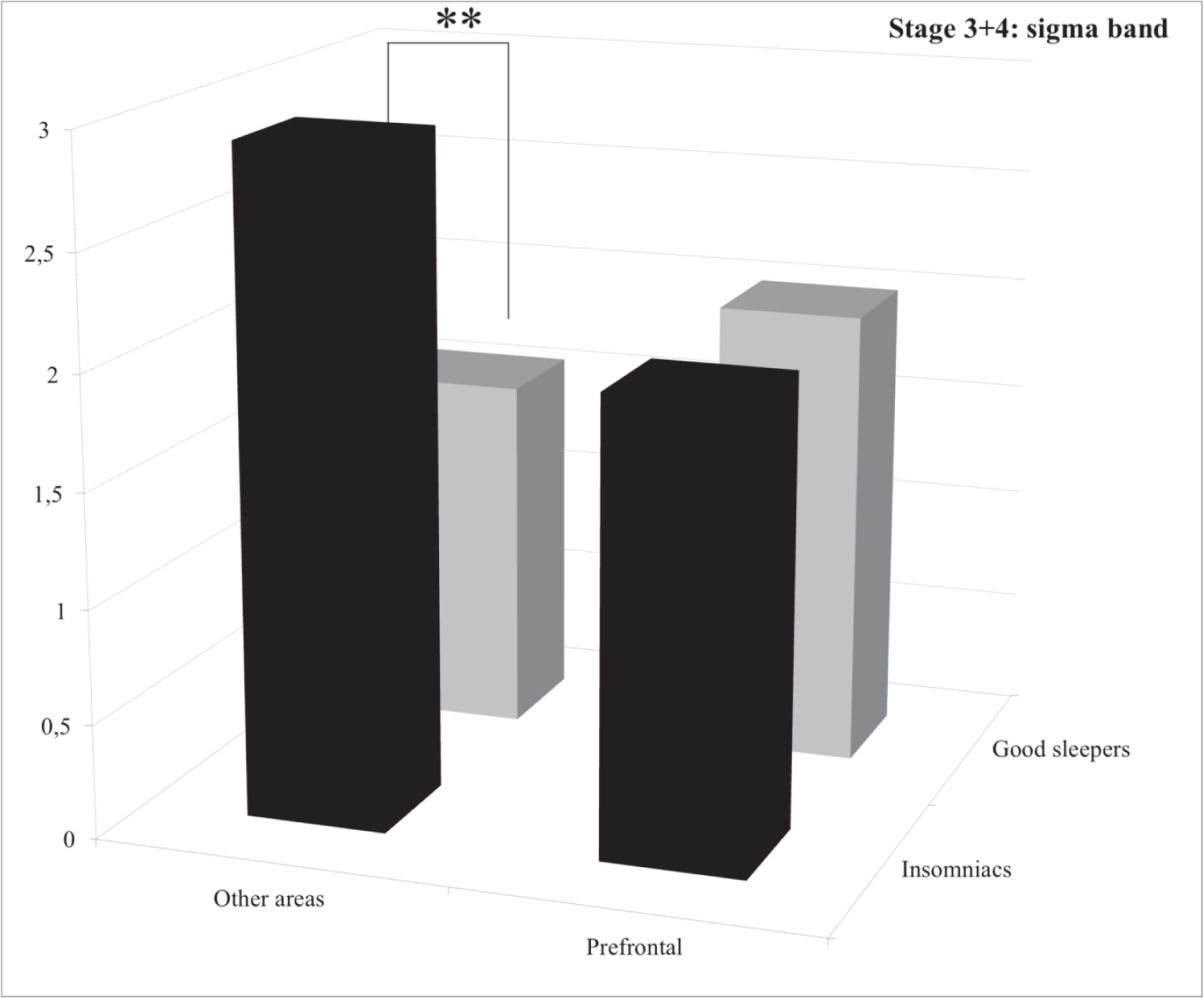
Relative power spectra for the sigma band during stage 3+4 for both groups (insomnia patients and good sleepers) and each areas (others vs prefrontal). ** p<0.05.

**Figure 5:**
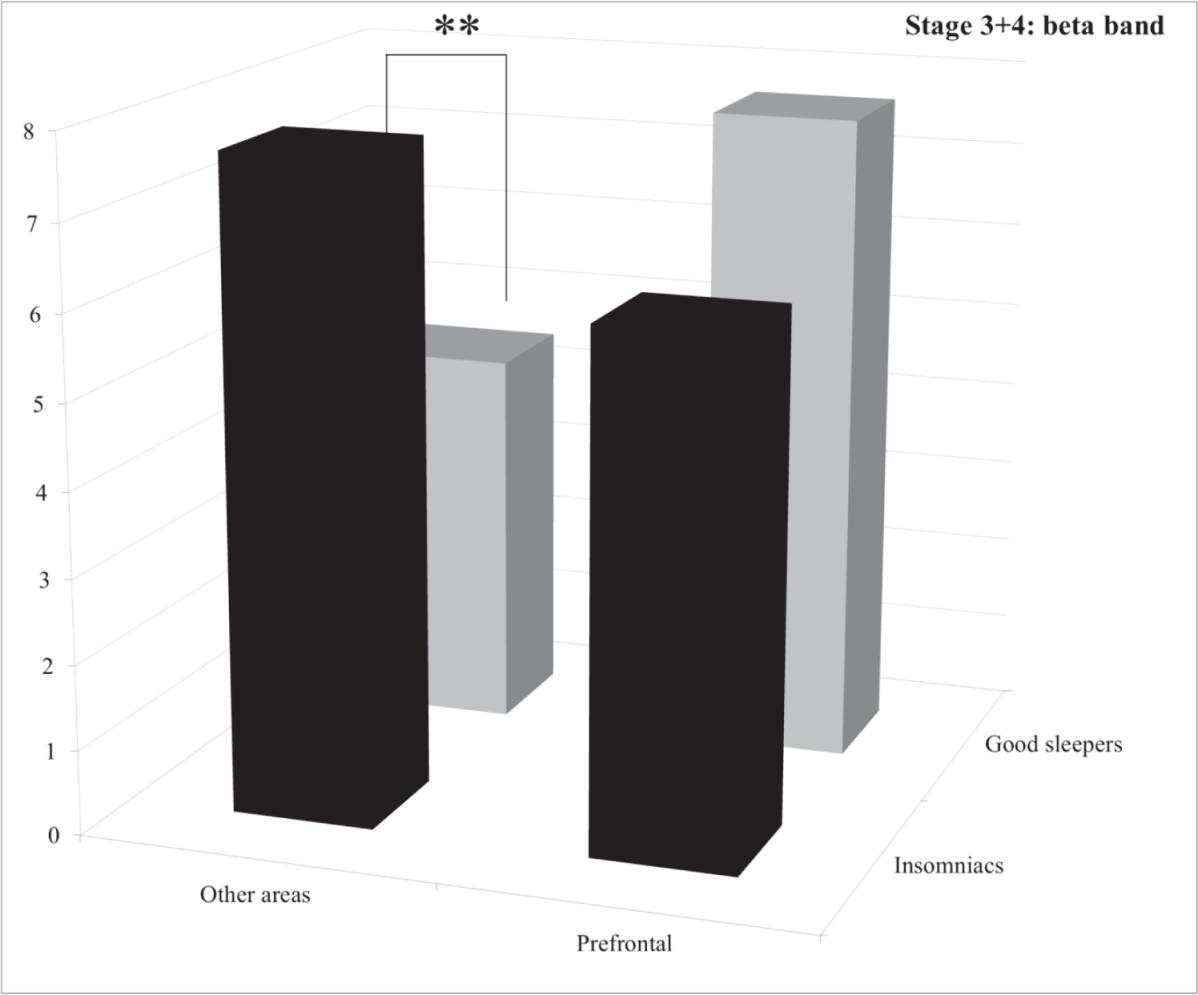
Relative power spectra for the beta band during stage 3+4 for both groups (insomnia patients and good sleepers) and each areas (others vs prefrontal). ** p<0.05.

**Stage 3+4.** Statistical results for the group effect revealed that PI patients displayed significantly more alpha and sigma power spectra than good sleepers and less delta power spectrum with a trend. For the delta band, post hoc comparisons of the significant group by area interaction revealed that PI patients displayed significantly less power spectrum than good sleepers in the “other areas” (*p* = 0.039) whereas no significant difference between groups was found for the prefrontal area (*p* = 1.0) (See Fig. 3). For the sigma band, post hoc comparisons of the significant group by area interaction revealed that PI patients displayed significantly more power spectrum in the “other areas” (*p* = 0.0056), whereas no significant difference between groups was found for the prefrontal area (*p* = 1.0) (See Fig. 4). For the beta band, post hoc comparisons of the significant group by area interaction revealed that PI patients displayed significantly more power spectrum in the “other areas” (*p* = 0.013), whereas no significant difference between groups was found for the prefrontal area (*p* = 0.38) (See Fig. 5).

**Figure 6:**
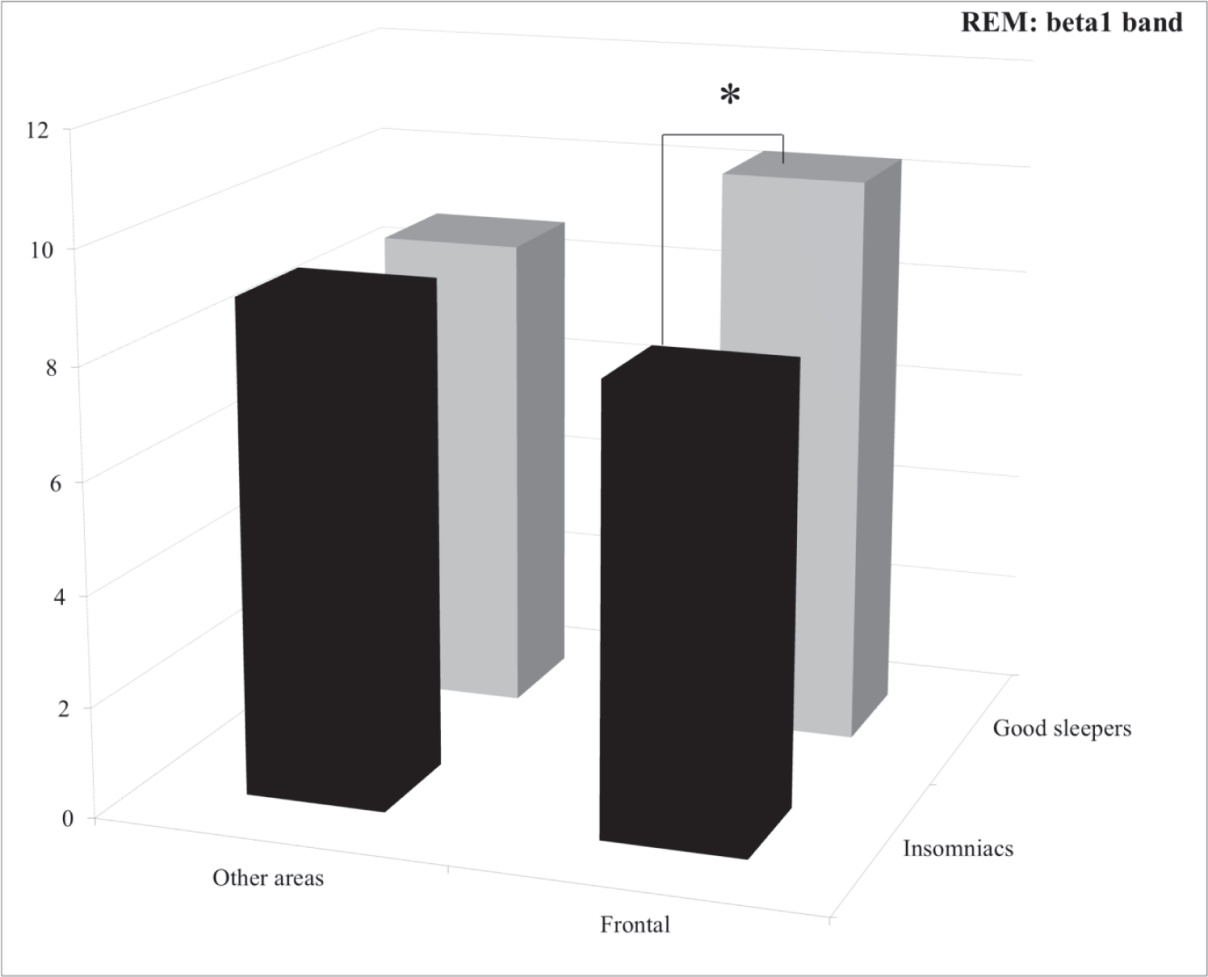
Relative power spectra for the beta1 band during stage REM for both groups (insomnia patients and good sleepers) and each areas (others vs prefrontal). *0.1>p>0.05.

**Stage REM.** For the beta1 band, post hoc comparisons revealed that the lower power spectrum of the PI patients compared to good sleepers in the prefrontal area was close to significant level (*p* = 0.060) (See Fig. 6).

## Discussion

The aim of the current investigation was to evaluate if, in PI patients, the prefrontal cortex displayed a specific sleep pattern compared to good sleepers. The main results were that i) the prefrontal cortical activity in PI patients differs from the one recorded in the other cortical areas; ii) this specific difference between cortical activity of areas in PI patients was observed both during the NREM and the REM sleep iii) the relative beta power spectrum in PI patients was higher compared to good sleepers for the other cortical areas and this result was true throughout the NREM sleep (stage 2 and stage 3+4).

The specific prefrontal activity in PI patients was revealed during the NREM sleep (stage 2 and 3+4) by a concomitant lack of difference between groups for the delta and beta power spectra in the prefrontal area with the presence of cortical activity differences between groups in other areas. We also found cortical activity difference between groups for the sigma band during the stage 3+4 in the other areas while no group difference was revealed for the prefrontal area. During the REM sleep, the only difference between both groups was found for the prefrontal electrodes, as we observed that PI patients displayed less beta1 power spectra than good sleepers in this cortical area. To our knowledge, this is the first report about a specific prefrontal sleep pattern compared to other cortical areas in PI patients.

Our NREM findings for the other areas (occipital, central and temporal areas) revealed that PI patients displayed i) more beta and less delta power spectra and ii) greater sigma power spectrum compared to good sleepers. These results are in agreement with previous reports [11–15,31]. Nevertheless, the present study is the first one that describes simultaneously an increased beta power spectrum throughout the entire NREM sleep (i.e stage 2 and stage 3+4) in PI patients. In is important to notice that our participants were free of medications since at least two years. Therefore, our results about an increase sigma power spectrum could not be linked to the so called “GABA-Benzodiazepine signature” which refers to suppression of frequencies below 10 Hz and an increase of sigma band power [32,33]. Our results can be interpreted as an hyperarousal state during the entire NREM sleep period in PI.

Concerning our REM results, we found a lower beta1 value in the prefrontal area in PI patients compared to good sleepers but no difference between groups in the other cortical areas (i.e central, temporal, occipital). The lack of difference in activity between groups in the central area in the current study is in line with the results of Spiegelhalder et al., [15]. Concerning our findings in the prefrontal area, they contrast with those of Merica et al., [11], who found increased beta and gamma bands (i.e higher frequency bands) activity in F4 in PI patients compared to good sleepers. However, the authors’ were not convinced by their REM results as they mentioned possible NREM sleep intrusions in the REM sleep leading to unexpected results (i.e increased in higher frequency bands) during this period.

The specific sleep pattern revealed in the prefrontal area compared to other cortical areas during the NREM and the REM stages in PI patients extends previous findings which revealed frontal modifications or alterations in PI during the waking state. Indeed, by using neuroimaging, previous studies showed that PI patients displayed i) a greater general brain metabolism during wake and sleep [34] ii) a reduced relative metabolism in the prefrontal cortex in comparison with other brain areas during the waking state [34], and iii) a specific hypoactivation of the prefrontal cortex during a letter fluency task in comparison to good sleepers [21].

Altogether, our NREM and REM results indicate that PI patients displayed a specific dysfunction in the reactivation of the limbic system during the REM period rather than a global prefrontal dysfunction during sleep. During the REM sleep, in the prefrontal cortex, there is a reactivation (i.e an increase of the cortical activity) of the so called “anterior paralimbic REM activation area” [35,36] while other parts of the prefrontal cortex remain deactivated [35,37]. In contrast, during the NREM sleep, the entire prefrontal cortex is deactivated (including the anterior paralimbic REM activation area). Our results may then be interpreted as follows: i) the hypoarousal reflected by less beta1 activity during the REM sleep in PI patients in prefrontal electrodes, may be linked to a problem in the reactivation of the anterior paralimbic area during the REM sleep; ii) the lack of hyperarousal reflected by no difference in beta activity between groups during the NREM sleep in prefrontal electrodes, may be linked to the fact that the entire prefrontal cortex is deactivated during this sleep stage.

Our results about a concomitant greater sigma and alpha power spectra during the NREM sleep in PI patients compared to good sleepers seem to support the idea according to PI patients displayed more sleep-protective mechanisms than good sleepers. The sleep microstructure modifications linked to PI and their interpretations are complex to understand and various results can be found in the literature (See, [10] for a review). Among them, a consistent finding in PI patients is an higher cognitive brain state during sleep. However, it is not clear whether PI patients displayed more sleep-promoting mechanisms compared to good sleepers [38,39]. The increased sigma power spectrum during stage 3+4 in insomnia patients compared to good sleepers found in the other areas can be view as an increased sleep stability [25]. Concerning the alpha rhythm, a recent review underlined its role in inhibition mechanisms by reflecting a “closed thalamic gate” [40]. Consequently, our findings suggest that PI had more sleep-promoting mechanisms (reflected by greater sigma and alpha rhythms) than good sleepers which can be linked to compensatory mechanisms against the hyperarousal state (reflected by greater beta rhythm).

This study has some limitations especially regarding our small sample size. However, the patients included were carefully diagnosed and had PI without comorbidity, and without any drugs intake. Complementary investigation should be interesting to perform in order to extend our findings. In addition, further investigations are also needed in other subtypes of insomnia in order to assess if the specific prefrontal activation pattern found in PI patients is also present in other insomnia subtypes. Such studies are needed to better understand the pathophysiology of insomnia. Also, the sleep recording has been performed during a single night of polysomnography. It is well known that participants’ sleep architecture during the first night in a sleep laboratory environment differs from subsequent night, which is called the first night effect (FNE). Especially, it has been shown than, in good sleepers, the TST, the time spent in REM sleep and the sleep efficiency are lower, and REM latency and the number of WASO are increased [41,42] compared to subsequent nights. In the current investigation, we assumed that the relative high amount of Stage 1 found in good sleepers is due to this FNE as it leads to more WASO. Concerning the insomnia patients group, the rather high amount of SWS found can be explained by the reverse first night effect (RFNE), already described in these patients [41,43]. Especially, it has been shown that insomnia patients sleep better at the sleep laboratory than at home [43]. Consequently, the SWS can be increased and thus here appeared unusually high for PI patients.

## Conclusion

To conclude, the present study gives for the first time evidence about a prefrontal sleep pattern that differs by far from the one in other cortical areas during both the NREM and the REM sleep. Altogether, our results suggest that PI patients displayed a specific dysfunction in the reactivation of the limbic system during the REM period. Our results give also additional arguments in favor of sleep-protection mechanisms displayed by PI patients at the meantime than a cortical hyperarousal.

## Supporting Information

S1 Table Relative power spectra for the delta band during stage 2. (XLSX)

S2 Table Relative power spectra for the beta band during stage 2. (XLSX)

S3 Table Relative power spectra for the delta band during stage 3+4. (XLSX)

S4 Table Relative power spectra for the alpha band during stage 3+4. (XLSX)

S5 Table Relative power spectra for the sigma band during stage 3+4. (XLSX)

S6 Table Relative power spectra for the beta band during stage 3+4. (XLSX)

S7 Table Relative power spectra for the beta1 band during stage REM. (XLSX)
